# *msp1, msp2,* and *glurp* genotyping to differentiate *Plasmodium falciparum* recrudescence from reinfections during prevention of reestablishment phase, Sri Lanka, 2014–2019

**DOI:** 10.1186/s12936-024-04858-6

**Published:** 2024-01-27

**Authors:** Kumudunayana T. Gunasekera, Risintha G. Premaratne, Shiroma M. Handunnetti, Jagathpriya Weerasena, Sunil Premawansa, Deepika S. Fernando

**Affiliations:** 1 Anti Malaria Campaign, Ministry of Health, 555/5 Public Health Complex, Elvitigala Mawatha, Colombo 5, Sri Lanka; 2 World Health Organization Regional Office for South-East Asia, New Delhi, India; 3 Institute of Biochemistry, Molecular Biology and Biotechnology, University of Colombo, Colombo, Sri Lanka; 4 Department of Zoology and Environmental Science, University of Colombo, Colombo, Sri Lanka; 5 Department of Parasitology, Faculty of Medicine, University of Colombo, Colombo, Sri Lanka

**Keywords:** Genotyping, *msp1*, *msp2*, *Glurp*, Recrudescence, Malaria prevention of re-establishment

## Abstract

**Background:**

Sri Lanka after eliminating malaria in 2012, is in the prevention of re-establishment (POR) phase. Being a tropical country with high malariogenic potential, maintaining vigilance is important. All malaria cases are investigated epidemiologically and followed up by integrated drug efficacy surveillance (iDES). Occasionally, that alone is not adequate to differentiate *Plasmodium falciparum* reinfections from recrudescences. This study evaluated the World Health Organization and Medicines for Malaria Venture (MMV) recommended genotyping protocol for the merozoite surface proteins (*msp1, msp2*) and the glutamate-rich protein (*glurp*) to discriminate *P. falciparum* recrudescence from reinfection in POR phase.

**Methods:**

All *P. falciparum* patients detected from April 2014 to December 2019 were included in this study. Patients were treated and followed up by iDES up to 28 days and were advised to get tested if they develop fever at any time over the following year. Basic socio-demographic information including history of travel was obtained. Details of the malariogenic potential and reactive entomological and parasitological surveillance carried out by the Anti Malaria Campaign to exclude the possibility of local transmission were also collected. The *msp1, msp2,* and *glurp* genotyping was performed for initial and any recurrent infections. Classification of recurrent infections as recrudescence or reinfection was done based on epidemiological findings and was compared with the genotyping outcome.

**Results:**

Among 106 *P. falciparum* patients, six had recurrent infections. All the initial infections were imported, with a history of travel to malaria endemic countries. In all instances, the reactive entomological and parasitological surveillance had no evidence for local transmission. Five recurrences occurred within 28 days of follow-up and were classified as recrudescence. They have not travelled to malaria endemic countries between the initial and recurrent infections. The other had a recurrent infection after 105 days. It was assumed a reinfection, as he had travelled to the same malaria endemic country in between the two malaria attacks. Genotyping confirmed the recrudescence and the reinfection.

**Conclusions:**

The *msp1, msp2* and *glurp* genotyping method accurately differentiated reinfections from recrudescence. Since reinfection without a history of travel to a malaria endemic country would mean local transmission, combining genotyping outcome with epidemiological findings will assist classifying malaria cases without any ambiguity.

**Supplementary Information:**

The online version contains supplementary material available at 10.1186/s12936-024-04858-6.

## Background

Sri Lanka eliminated indigenous malaria in 2012, and was certified as a malaria free country in 2016. The conducive tropical environment still facilitates the perennial breeding of the major vector of malaria, *Anopheles culicifacies*, in many parts of the country [1]. Therefore, the country is at high risk of re-establishment of the disease [2]. This was aptly demonstrated by the introduced malaria case diagnosed in 2018 [3]. The introduction of the urban invasive vector species *Anopheles stephensi* a few years ago has increased receptivity [4]. The Anti Malaria Campaign (AMC), in line with the national strategic plan for malaria, is taking measures to prevent the re-establishment of malaria and prevent deaths due to malaria [5]. As a requirement, quality-assured diagnostic service is maintained in the country. Passive case detection is carried out comprehensively. In addition, the AMC is conducting targeted proactive case detection among clusters of high-risk individuals based on the importation risk [6]. Currently, approximately 50 imported malaria cases are reported each year [7]. Each reported case is fully investigated and followed up according to integrated drug efficacy surveillance (iDES) [8, 9]. Reactive parasitological and entomological surveillance is conducted based on the history of travel and the possibility of local transmission [10]. The findings are reviewed by an expert panel, the Case Review Committee of the AMC before the case is classified as imported, indigenous, introduced, relapse, or recrudescence [11]. As a country in the POR phase any recurrent infection classified as a reinfection, in a patient that has no history of overseas travel to a malaria-endemic country, would be an introduced case or an indigenous case. As this means the resumption of local transmission, comprehensive interventions are needed to be done immediately. If a recurrent infection is classified as a recrudescence, the possibility of treatment failure needs to be considered, and there is a need to re-visit the anti-malarial treatment guidelines. Although reactive surveillance often provides the answer, this requires a lot of field activities and resources. Occasionally, that alone is not adequate to conclude the case classification with certainty, especially when there is a long duration between the initial and recurrent infection.

Genotyping in identifying introduced and indigenous malaria cases in countries that have eliminated malaria has been well documented [12]. To distinguish recrudescence (true treatment failure) from a reinfection of *Plasmodium falciparum,* a cost-effective PCR genotyping protocol has been recommended by the World Health Organization (WHO) and Medicines for Malaria Venture (MMV) [13, 14]. Based on length polymorphic genes encoding the merozoite surface proteins (*msp1* and *msp2*) and the glutamate-rich protein (*glurp*), this enabled application of genotyping even in settings with limited resources. Recently, this method has been criticized as underestimating true drug failure rates in certain epidemiological conditions [15–18] and the WHO has published revised guidelines recommending the use of microsatellites instead of *glurp* for low to moderate and high transmission settings in Africa, while outside Africa the previous protocol of the *msp1, msp2* and *glurp* genotyping is still applicable [19]. As a country in the POR phase with imported malaria cases originating from countries with varying endemicities, any misleading interpretation would have a detrimental impact on the malaria-free status of Sri Lanka. This study assessed the relevance and the role of the *msp1, msp2* and *glurp* genotyping procedure to differentiate *P. falciparum* recrudescence from reinfections in the POR phase in Sri Lanka.

## Methods

### Study population and sample collection

All *P. falciparum* patients detected from April 2014 to December 2019 at the central laboratory of the AMC, were considered for this study. They were treated as inward patients with artemether–lumefantrine (AL) and a single dose of primaquine, according to national malaria treatment guidelines of the Ministry of Health, Sri Lanka [9]. All were followed up according to iDES irrespective of any inclusion or exclusion criteria, daily on D1, D2, D3, and thereafter on a weekly basis for up to 28 days [20]. In addition, patients were advised to get tested for malaria if they develop a fever at any time over the following year.

On the day of diagnosis (D0) and all follow-up days, thick and thin blood smears were prepared and parasitaemia was assessed by Giemsa-stained microscopy [21]. In addition, 125 µL of blood was collected to Flinders Technology Associates (FTA) filter paper cards to be used for genotyping and nested PCR confirmation, after obtaining informed consent. FTA cards were air-dried and placed in individual zip-lock plastic bags containing silica gel and stored at room temperature. Parasite DNA was extracted from the FTA cards using the QiaAmp DNA blood mini kit (Qiagen, Germany). Briefly, three dried blood spots of 3 mm diameter were used from the FTA cards and the spin protocol was followed according to the manufacturer’s instructions. Extracted DNA was eluted in 200 µL of nuclease free water and stored at − 80 °C until further analysis. All blood smears from D7 onwards were confirmed by nPCR [22]. If a patient was diagnosed with a recurrent *P. falciparum* infection, the parasite species was confirmed by nPCR.

### Clinico epidemiological characteristics

Basic socio-demographic information including the history of travel was obtained. Details on the malariogenic potential and the findings of the reactive entomological and parasitological surveillance carried out by the AMC to exclude the possibility of local transmission was also collected.

### Classification of recurrent infections

This was done considering the outcome of iDES and detailed case investigations conducted including reactive entomological and parasitological surveillance. A recurrent *P. falciparum* asexual parasitaemia within 28 days was classified as a late clinical failure (LCF) or a late parasitological failure (LPF) according to the WHO-specified criteria [13].

For recurrent infections that were detected after 28 days, to make the decision, the duration between the two infections was also considered. The possibility of an imported reinfection was also considered if there is evidence of travel to a malaria-endemic country. In the absence of a history of travel, the possibility of local transmission was considered.

### Genotyping of *msp1*, *msp2*, and *glurp *for *P. falciparum* infection

In any patient who presented with a recurrent infection, genotyping of both initial and recurrent samples was carried out to determine whether the recurrent infection is a reinfection or a recrudescence. Genotyping of polymorphic regions from *P. falciparum* merozoite surface proteins (*msp1* and *msp2*) and glutamate-rich protein (*glurp*) coding sequences was carried out according to the standardized protocols recommended by the WHO and MMV [13, 14]. All assays were performed using a BIO-RAD T100™ Thermal cycler. For the amplification of *msp1* and *msp2,* initially, a single multiplex primary PCR assay was performed. This was followed by two separate family-specific nested PCR assays to determine the presence of 3D7 and FC27 allelic families in the central polymorphic region of *msp2*. To detect the three allelic families in block 2 of *msp1* (namely, K1, MAD20, and RO33 allelic families) three separate nested PCR assays were performed. For *glurp*, a separate primary PCR was performed followed by a nested PCR. Each polymorphic domain was amplified in 20 µL reaction mixture containing 1 µL of DNA, 0.3 µM of each primer, 2 mM MgCl_2_, 200 µM of each dNTP, and 1 U DNA polymerase (AmpliTaq, Applied biosystems). The primer sequences and the parameters of the PCR assays are given in Additional file 1: Table S1 and Additional file 2: Table S2 respectively. PCR amplicons were separated and visualized on 2% agarose gel stained with ethidium bromide. PCR assays were repeated if any allelic family or positive controls were negative. The 3D7 and Dd2 isolates were used as positive controls.

### Interpretation of the genotype patterns

For each of the different allelic family (ies) of *msp1, msp2,* and *glurp* genes, the genotypes were analysed by comparing the genotype/allele pattern of the initial and the recurrent samples. Interpretation of the genotyping outcome was done according to the procedure described by Felger and Snounou [14]. The pair of amplified products of the initial and the recurrent infections of one patient was run on agarose gel electrophoresis side by side for each of the different allelic families of *msp1, msp2,* and *glurp*. The comparison of PCR fragments was performed by two independent readers and the size of the DNA fragments was estimated based on visual inspection using a 100 bp DNA ladder marker. Fragment sizes were defined in an enlarged gel picture. Amplified fragments were considered to be different between initial and recurrent samples if the sizes of the bands differed by more than 20 bp for *msp1* and *msp2* and more than 50 bp for *glurp*. If all or any one of the allele in an allelic family is shared between the two samples, the outcome for the gene was interpreted as a recrudescence. A reinfection was indicated when all alleles (for any marker gene) of the initial and the recurrent samples were completely different. If a reinfection is identified with any marker gene, the overall outcome is a reinfection regardless of the result of the other allelic families.

## Results

### Findings of the case investigations and follow-up

Among the 106 imported *P. falciparum* infections detected, six had recurrent *P. falciparum* infections. The Clinico-epidemiological characteristics including the findings of reactive surveillance and the case classification of these six patients are given in Table 1. Five of these recurrent infections (patient numbers 1–5), occurred within the 28 days follow-up period of the iDES (D15 to D28). The initial parasite densities ranged from 12,286 parasites/µL to 139,144 parasites/µL and parasitaemia cleared within 3 days. In these five patients, the parasite count on the day of recurrent parasitaemia was lower than the initial parasite count (D0). Except for the fifth patient who was treated with Dihydroartemisinin/piperaquine (DHA PPQ), all others were treated again with a full course of AL, and parasitaemia cleared within 2 days. There was no history of travel to a malaria-endemic country between the initial and recurrent infection. Reactive entomological surveillance carried out indicated that the receptivity risk for the primary malaria vector in Sri Lanka, *An. culicifacies* was low to moderate. While the parasitological surveillance indicated high importation risk in relevant areas, there was no evidence of ongoing local transmission to assume that the patient had contracted these infections locally. Therefore, these recurrent infections were classified as recrudescence.

**Table 1:** Clinico-epidemiological characteristics and classification of treatment outcome of patients with recurrent infections

Patient Number	1	2	3	4	5	6
Age	46	35	30	40	40	46
Gender	F	M	M	M	M	M
Initial infection						
History of travel	Congo	Mozambique	Madagascar	Uganda	Congo	Central African Republic (CAR)
Days from the onset of symptoms to diagnosis	7*	1	3	7	5	27*
Days between arrival to diagnosis	5	9	7	13	14	11
Parasitaemia (No. of parasites/µL)	73,098	12,286	139,144	25,800	84,400	176
Clearance of parasitaemia	D1	D1	D2	D2	D2	D1
Whether reactive surveillance conducted	Yes	Yes	Yes	Yes	Yes	Yes
Recurrent infection						
Re appearance of parasitaemia	D16	D19	D19	D28	D18	D105
Parasitaemia (µL^−1^)	64	7,640	6,613	14,145	13,200	1,415
Clearance of parasitaemia	D1	D1	D2	D2	D2	D2
History of travel	No	No	No	No	No	Yes (CAR)
Risk of importation	High	High	High	Moderate	High	High
Receptivity in the area	Low	Low	Low	High	Low	Moderate
Whether reactive surveillance conducted	Only travel contacts screened	Only travel contacts screened	Only travel contacts screened	Only travel contacts screened	Both travel and geographical contacts	Both travel and geographical contacts
Classification of recurrent infection	LCF/recrudescence	LCF/recrudescence	LCF/recrudescence	LCF/recrudescence	LCF/recrudescence	Reinfection

*These patients had onset of symptoms before the arrival to the country

The other recurrent infection (patient number 6) was detected after 105 days in a patient who had an initial *P. falciparum* infection with a parasitaemia of 176 parasites/µL. As the initial parasitemia in this patient cleared in two days and remained negative during the 28 days of the iDES, the initial infection was considered an adequate clinical and parasitological response (ACPR). Case investigation of the recurrent infection revealed that this patient had re-visited the same malaria-endemic country in Africa (Central African Republic) between the first infection and the recurrent infection and the reactive surveillance did not find evidence of local transmission. Therefore, the recurrent infection was assumed to be a reinfection contracted during his second visit to the Central African Republic. However, the possibility of persistent parasitaemia and recrudescence had to be excluded.

### Results of genotyping of initial and recurrent infections

**Fig. 1:**
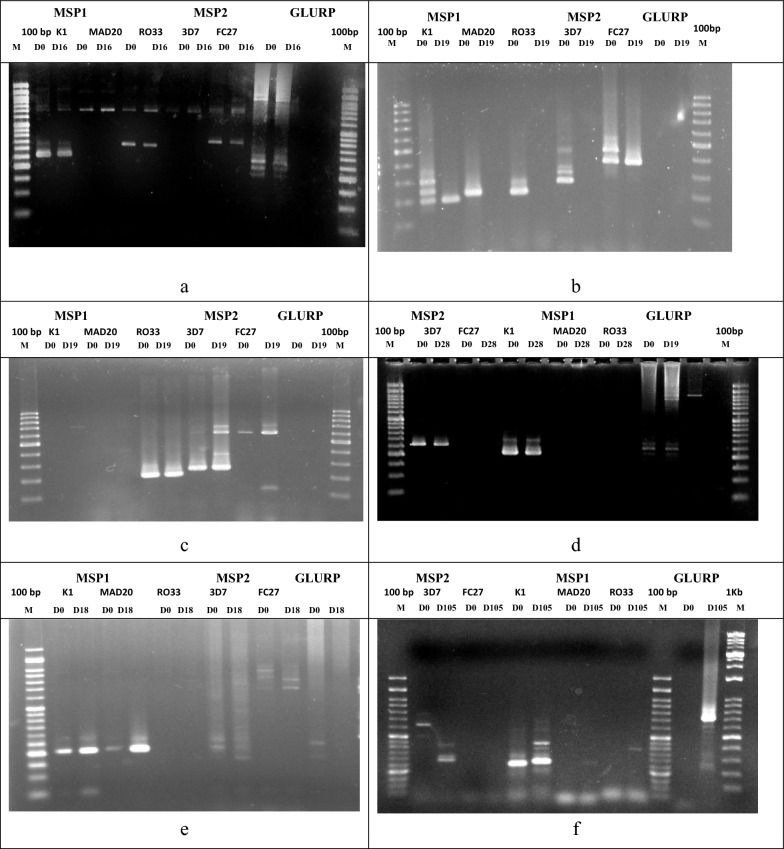
Photograph showing the resolution of amplified products of *msp1, msp2,* and *glurp* allelic families of the six patients with recurrent infections. **a** Patient 1 had identical *msp1-*K1, *msp1*-RO33, *msp2-*FC27, and *glurp* alleles. **b** Patient 2 had alleles of all *msp1* and *msp2* allelic families in the initial sample, while the recurrent sample had only an *msp1-*K1 allele and an *msp2-*FC27 allele. **c** The recurrent infection of patient 3 had identical *msp1-*RO33 and *msp2*-3D7, and *msp2*-FC27 alleles and additional new *msp2*-FC27 alleles. **d** Patient 4 shared *msp1-*K1, *msp2*-3D7, and shared *glurp* in the initial and recurrent samples. **e** Patient 5 has similar *msp1-* K1 and *msp1-*MAD 20 alleles. Shared alleles were seen in *msp2*-FC27 and *msp2*-3D7. *Glurp* fragments were present in both samples. **f** Patient 6 had *msp2*-3D7 and *msp1-*K1 alleles in the initial sample while the recurrent sample on D105, had different *msp2*-3D7 and K1 alleles and faint bands of additional *msp1-*RO33 and *msp1-*MAD20 alleles indicating a reinfection

**Fig. 2:**
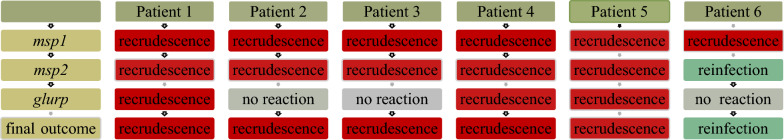
Details of the outcome of *msp1*,* msp2*, and *glurp*, genotyping of the initial and recurrent *P. falciparum* isolates obtained from the patients who had recurrent infections

In this study all three genes (*msp1, msp2* and *glurp*) were genotyped for comparison. Amplification of *msp1* and *msp2*, was successful in all initial and recurrent infections, while *glurp* was amplified only in three patients. In all instances positive controls were amplified. For all target genes amplified, the initial infections showed a high multiplicity of infection (Fig. 1), indicating probable exposure to high endemicity. Recurrent infections showed a varying degree of multiplicity of infection. Comparison of initial and recurrent genotypes showed that in patients1–5, same alleles were shared between initial and the recurrent infections indicating recrudescence (Fig. 1a–e). Patient 6 had different alleles for the *msp2* gene amplified in the recurrent infection indicating a reinfection (Fig. 1f). The details of the genotyping outcomes of these six patients are given in Table 2, while Fig. 2 gives a comparison of the outcome of the 3 genes.

**Table 2:** Observed allele fragment lengths of the initial and recurrent infections of the six patients

Patient no	Genotype/alleles	*msp1*			*msp2*		*glurp*	Genotyping outcome & Classification of recurrent infection
		K1	MAD20	RO33	FC27	3D7		
1	D0	280		350	400	–	200, 250, 280, 800	LCF/recrudescence (compatible with the epidemiological findings)
	D16	280		350	400		200, 250, 280, 800	
2	D0	200, 250, 300	250	200, 230	250, 300, 350	400, 450, 500	Not amplified	LCF/recrudescence (compatible with the epidemiological findings)
	D19	200	–	*–*	–	400	Not amplified	
3	D0	–	–	230	300, 400, 600, 700	600	Not amplified	LCF/recrudescence (compatible with the epidemiological findings)
	D19	–	–	230	300, 400, 600, 700	150, 600, 700	Not amplified	
4	D0	230, 250	*–*	–	–	250	200, 250, 300	LCF/recrudescence (compatible with the epidemiological findings)
	D28	230, 250	–	–	–	250	200, 250, 300	
5	D0	200	200	*–*	700, 800, 900	200, 250,	*glurp*	LCF/recrudescence (compatible with the epidemiological findings)
	D18	200	200	*–*	700, 800	150, 200, 250	*glurp*	
6	D0	280, 350	–	–	–	650	Not amplified	All alleles different on D0 and D105 for *msp2* Reinfection (compatible with the epidemiological findings)
	D105	300, 350, 400, 450	280	400	–	280, 400	700, 800

## Discussion

As a country that has eliminated malaria, Sri Lanka needs to prevent re-establishment of malaria transmission in the country. This requires immediate preventive measures for any imported, introduced, or indigenous case. Considering the *P. falciparum* artemisinin partial resistance, immediate identification of recrudescence from reinfections is crucial.

Unlike in therapeutic efficacy studies in endemic settings, where “molecular correction” by genotyping is used to guide the use of anti-malarials, obtaining evidence for the malaria-free status was an objective of this study. Hence, the genotyping outcome was compared with epidemiological findings. The six patients with recurrent infections had a history of travel to African countries. Genotyping revealed highly polymorphic genotypes in primary infections of these recurrent infections, indicating that they have been initially exposed to high transmission settings. Heavy multiple infectious bites per person are common in sub–Saharan African countries with high transmission intensity [23, 24]. Thus, the African origin of these infections with recurrent attacks correlates well with the genotyping findings.

PCR is used to differentiate between reinfection and recrudescence by comparing the allelic variants present in the initial and recurrent samples. In high transmission settings, when patients harbour high multiplicity of infections or common genotypes, it may be difficult to differentiate reinfections from recrudescence. In such settings, *P. falciparum* reinfections as early as day 14 are known to occur [25]. Other studies have shown persisting asexual parasitaemia with more than 25% prevalence even after 14 days [26]. Depending on the prevalence of allelic variants in a particular region, there is always a possibility of a reinfection with the same genotypes that are in the patient before treatment. With the recent recommendations for advanced genotyping procedures for African countries [19], it will be important to determine whether the genotyping protocol used in this study would relate to the epidemiological findings in differentiating recrudescence from reinfections and thereby provide evidence to maintain malaria-free status.

Methodologies used for genotyping *P. falciparum* have their advantages and limitations. Capillary electrophoresis (CE) has been recommended for precise fragment sizing for genotyping especially in high transmission settings [27]. However, CE may not be readily available in resource-limited settings. In such situations, the limitations of the *msp1, msp2* and *glurp* genotyping procedure need to be properly assessed especially when applying the findings for decision-making.

This study was done in a setting where local transmission has been eliminated and selective control measures were continued in the malaria POR phase. Since the resources were limited, agarose gel electrophoresis was performed and fragment sizes were determined manually which resulted in five recrudescence and one reinfection. It is known that the limited resolution of this method (where genotypes differing in less than 20 bp for *mps1* and *msp2* and less than 50 bp for *glurp* are considered as one) can be a major factor in the overestimation of the treatment failure rate or in some instances mis-classifying recrudescences as new infection [15, 28, 29]. However, the findings of this study compared well with the epidemiological findings indicating the applicability of genotying protocol used.

Yet, the limitations of this genotyping procedure need to be properly identified and assessed. Amplification bias is known to suppress long fragment alleles and preferentially amplify short fragments thereby compromising the detection of co-infecting clones [17]. In high transmission settings, the range of MOI has been reported from a single strain to more than 10 strains in an isolate. In such multi-clonal infections, in the presence of a dominant clone, the minority clones consisting a small proportion of biomass might fall below the detection limit of the genotyping method. Such cryptic minor variants can be missed by PCR- based detection due to completion for primer or other constituents of the reaction mix by the more abundant clones that may be present in a patient blood [15–17, 28–33]. Due to this imperfect clone detectability, variants that would be missed in the initial genotyping would later appear at a detectable level in the recurrent parasitaemia (resistant) and there is a possibility of misidentifying such a true recrudescence as a false reinfection. In this study too, the observation of one or more extra alleles of *msp1* and *msp2* genotyping in post-treatment samples in some of the patients is a good example for this phenomenon. However, as was evident in this study, the WHO definition of a recrudescence, that is the presence of at least one shared genotype in the compared pre and post-treatment samples at all loci seems to be an effective method that would overcome most of the above limitations [13]. In the absence of local transmission, (as confirmed by the reactive entomological and parasitological surveillance) this confirms that the infections are actual recrudescences. This highlights the accuracy of *msp1, msp2* and *glurp* genotyping method in categorizing recurrent infections of imported malaria cases in the POR phase in Sri Lanka. Furthermore, the outcome of genotyping confirmed the epidemiological finding which indicated that these patients have not travelled out of the country in between the initial and recurrent infection and that there was no evidence for local transmission.

Persisting parasitaemia after 42 days post-treatment have been reported among imported malaria cases in malaria-free settings [34]. For countries with high malariogenic potential such as Sri Lanka, such persisting parasitaemia and recrudescence would result in extensive reactive surveillance and control measures. Therefore, it would be important to find out with certainty whether such a recurrent infection is a recrudescence or a reinfection. In fact, the WHO recommends genotyping to confirm all reinfections and recrudescence that occur after 28 days [35]. In this study, genotyping confirmed that the recurrent infection detected after a lapse of 3 months (105 days) was a reinfection. Unlike in malaria endemic countries in the POR phase with high malariogenic potential, it is important to find out whether this reinfection is due to local transmission or due to a recent travel to a malaria endemic country. Since either genotyping or epidemiological findings alone may not provide conclusive evidence, combining the genotyping outcome with epidemiological findings will help to determine the origin of the infection.

According to the *msp1, msp2* and *glurp* genotyping procedure, if any marker shows only new alleles, the recurrent infection is considered a reinfection. This algorithm had been criticized as it had consistently underestimated true failure rates [17, 29] and obtaining consensus of two genotypes (2/3 algorithm) has been suggested by some researchers [27]. For the recrudescences analysed in this study, both methods would have given the same result. These criticisms were mainly due to the unreliable nature of *glurp*. This was also seen in this study, where *msp1* and *msp2* markers performed well, but *glurp* genotyping failed even after repeated testing in 50% of the samples. It may be assumed that the cause for this poor performance may not be an issue of the DNA template, or low copy number in the initial samples since *glurp* was not amplified even in samples with high parasitaemia, and when other genes have been amplified. Also other intrinsic factors could be a cause for this non amplification. Considering the unreliable nature of *glurp,* the WHO has recommended replacing *glurp* with a microsatellite marker for low to moderate and high transmission settings in Africa. For countries outside Africa, the current method is still applicable [19].

The information on the drug efficacy of antimalarials available in countries like Sri Lanka, where treatment outcomes can be observed without repeat inoculations from infectious mosquitoes would be useful for the status of drug resistance in malaria-endemic countries. However, it is important to note that treatment failure due to resistance to the ACT is only one of the possible reasons for recrudescence. Other possibilities like defects in absorbance and metabolism of drugs also need to be considered [36].

With zero indigenous disease burden, prompt case detection by health institutions, and healthcare providers is a major challenge as malaria is low in the differential diagnosis of patients presenting with fever. In some instances, this has resulted in unacceptable delays in diagnosis, sometimes even exceeding 30 days since the onset of fever [7]. In addition to the harmful effect on the patient, there is always a possibility that such a delay can result in the re-establishment of local transmission in Sri Lanka. This requires reactive surveillance to be carried out especially where the risk of importation and receptivity is high. Obtaining confirmatory evidence for the absence of local transmission as seen for these recurrent infections is important. In this study, the genotypically confirmed reinfection had a history of traveling to a malaria-endemic country in between the two infections. This indicates that the reinfection was contracted in that country. Since reinfection without a history of travel to a malaria-endemic country would mean local transmission, this highlights the importance of combining the genotyping outcome with the findings of the case investigation and reactive surveillance to ensure the malaria-free status.

In this study, the genotyping of initial and recurrent infections were performed when a recurrent infection was detected. Therefore all PCR assays were performed before revision of the WHO guidance in 2021. Since even according to the revised guidelines, *msp1, msp2* and *glurp* genotyping is applicable for countries outside Africa the findings of this study may be a good example for countries eliminating malaria or in the POR phase. In this context it is important to note that that *msp1, msp2* and *glurp* genotyping protocol can be applied even when resources are limited as it does not require expensive equipment and is not labour intensive.

## Conclusion

The *msp1, msp2,* and *glurp* genotyping differentiated recrudescence from reinfections and collaborated well with the epidemiological findings. Since reinfection without a history of travel to a malaria-endemic country would mean local transmission, combining genotyping outcomes with epidemiological findings will assist classifying malaria cases without any ambiguity.

### Supplementary Information

**Additional file 1: Table S1.** Primer sequences used for genotyping *P. falciparum*.

**Additional file 2: Table S2.** Reaction parameters of the genus and species-specific nested PCR reactions.

## Data Availability

The datasets generated and/or analyzed during this study are included in this published article.

## Abbreviations

ACPR: Adequate Clinical and Parasitological Response
AL: Artemether Lumefantrine
AMC: Anti Malaria Campaign
CE: Capillary electrophoresis
DHA PPQ: Dihydroartemisinin/piperaquine
GLURP: Glutamate Rich Protein
FTA: Flinders Technology Associates
iDES: Integrated Drug Efficiency Surveillance
LCF: Late Clinical Failure
LPF: Late Parasitological Failure
MMV: Medicines for Malaria Venture
MSP1: Merozoite Surface Protein 1
MSP2: Merozoite Surface Protein 2
nPCR: Nested Polymerase Chain Reaction
POR: Prevention of Reestablishment
WHO: World Health Organization
