# Supplementary material for: msp1, msp2, and glurp genotyping to differentiate Plasmodium falciparum recrudescence from reinfections during prevention of reestablishment phase, Sri Lanka, 2014–2019
Source: Malar J. 2024 Jan 27;23:35. doi: 10.1186/s12936-024-04858-6 (PMC10821543; doi:10.1186/s12936-024-04858-6)
Supplement: Supplementary file 1 — Additional file 1: Table S1. Primer sequences used for genotyping P. falciparum. [file 12936_2024_4858_MOESM1_ESM.docx]

Table S1. Primer sequences used for genotyping *P. falciparum*

| **PCR** | **Primer** | **Sequence (5’ – 3’)** |
| --- | --- | --- |
| *msp1* | M1-OF | CTAGAAGCTTTAGAAGATGCAGTATTG |
|  | M1-OR | CTTAAATAGTATTCTAATTCAAGTGGATCA |
| *msp2* | M2-OF | ATGAAGGTAATTAAAACATTGTCTATTATA |
|  | M2-OR | CTTTGTTACCATCGGTACATTCTT |
| family specific semi nested msp2 | S1fw | GCTTATAATATGAGTATAAGGAGAA |
|  | M5rev | GCATTGCCAGAACTTGAA |
|  | N5rev | CTGAAGAGGTACTGGTAGA |
| *msp1-* K1 allelic family | M1-KF | AAATGAAGAAGAAATTACTACAAAAGGTGC |
|  | M1-KR | GCTTGCATCAGCTGGAGGGCTTGCACCAGA |
| *msp1-*MAD20 allelic family | M1-MF | AAATGAAGGAACAAGTGGAACAGCTGTTAC |
|  | M1-MR | ATCTGAAGGATTTGTACGTCTTGAATTACC |
| *msp1-*RO33 allelic family | M1-RF | TAAAGGATGGAGCAAATACTCAAGTTGTTG |
|  | Ro33-R2 | CAAGTAATTTTGAACTCTATGTTTTAAATCAGCGTA |
| primary PCR *glurp* | G-F3 | ACATGCAAGTGTTGATCCTGAAG |
|  | G-F4 | TGTAGGTACCACGGGTTCTTGTGG |
| nested PCR *glurp* | G-NF | TGTTCACACTGAACAATTAGATTTAGATCA |
|  | G-F4 | TGTAGGTACCACGGGTTCTTGTGG |
