# Supplementary material for: msp1, msp2, and glurp genotyping to differentiate Plasmodium falciparum recrudescence from reinfections during prevention of reestablishment phase, Sri Lanka, 2014–2019
Source: Malar J. 2024 Jan 27;23:35. doi: 10.1186/s12936-024-04858-6 (PMC10821543; doi:10.1186/s12936-024-04858-6)
Supplement: Supplementary file 2 — Additional file 2: Table S2. Reaction parameters of the genus and species-specific nested PCR reactions. [file 12936_2024_4858_MOESM2_ESM.docx]

Table S2. Reaction parameters of the genus and species-specific nested PCR reactions

|  |  | *msp1&2* primary multiplex and *glurp* primary PCR | *msp2* family specific  nested PCR | *msp1* family specific nested PCR and *glurp* nested PCR |
| --- | --- | --- | --- | --- |
| Cycle |  | Temperature  & Time | Temperature  & Time | Temperature  & Time |
| Initial Denaturation | 1 | 94 ºC for 2 min. | 94 ºC for 2 min. | 94 ºC for 2 min |
| Denaturation | 30 cycles | 94 ºC for 30 sec. | 94 ºC for 30 sec | 94 ºC for 30 sec |
| Annealing |  | 54 ºC for 1 min | 50 ºC for 45 sec | 59 ºC for 1 min |
| Extension |  | 72 ºC for 1 min | 70 ºC for 90 sec | 72 ºC for 1 min |
| Final extension | 1 | 72 ºC for 5 min | 70 ºC for 5 min | 72 ºC for 5 min |
